# Investigating the Alleviating Effect of Fucoidan from *Apostichopus japonicus* on Ulcerative Colitis by Mice Experiments and In Vitro Simulation of Human Fecal Fermentation

**DOI:** 10.3390/foods14040574

**Published:** 2025-02-09

**Authors:** Lingyan Xue, Yuchen Huan, Yaoguang Chang, Yuming Wang, Qingjuan Tang

**Affiliations:** College of Food Science and Engineering, Ocean University of China, Qingdao 266400, China; xuelingyan31@163.com (L.X.); huanyuchen79@outlook.com (Y.H.); changyg@ouc.edu.cn (Y.C.); wangyuming@ouc.edu.cn (Y.W.)

**Keywords:** fucoidan from *Apostichopus japonicus*, inflammatory bowel disease, ulcerative colitis, gut microbiota

## Abstract

Background: Fucoidan from *Apostichopus japonicus* (Aj-FUC) is a marine polysaccharide extracted from the high-quality sea cucumber, which has received increasing attention for its multiple biological activities. Methods: In this study, Aj-FUC was extracted, and its basic structure was characterized, while the alleviating efficacy of Aj-FUC on ulcerative colitis (UC) was investigated using C57BL/6 mice. The improvement of Aj-FUC on the fecal gut microbiota in healthy individuals and inflammatory bowel disease (IBD) patients was explored using in vitro simulated fecal fermentation. Results: The results reflected that Aj-FUC treatment attenuated the histopathological damage associated with colitis, reduced the levels of IL-6, IL-1β, and TNF-α. Aj-FUC treatment also upregulated the expression of ZO-1 and occludin, thereby aiding in the repair of the intestinal barrier. Furthermore, Aj-FUC enhanced the levels of short-chain fatty acids (SCFAs) and helped restore the balance of gut microbiota, particularly by increasing the relative abundance of *Akkermansia*. In vitro simulation of fecal fermentation showed that Aj-FUC could modulate the gut microbiota of IBD patients and increase the relative abundance of beneficial bacteria. Conclusions: In conclusion, this study highlights that Aj-FUC can alleviate UC by modulating the levels of inflammatory factors, improving the intestinal barrier, and regulating the intestinal flora in a variety of ways.

## 1. Introduction

Inflammatory bowel disease (IBD) is an inflammatory disease of the intestinal tract of undetermined etiology, mainly categorized as ulcerative colitis (UC) and Crohn’s disease (CD) [1], and has been called the “Green Cancer” because of the difficulty in eradicating the disease [2]. Although the exact mechanisms that trigger inflammatory bowel disease are poorly understood, the interplay of host microbiota dysbiosis [3], environmental factors [4], and genetic background [5] is relevant to its pathogenesis. Surveys indicate that IBD was initially observed in developed Western countries, with a high incidence rate in Europe, North America, and Canada. In recent years, its incidence rate in developing countries such as Asia has maintained a continuous growth. IBD has evolved into a global health issue that now affects about 6–8 million people worldwide. Once someone develops IBD, it can cause a great deal of pain and burden to the patient and their family.

Currently, the treatment of UC mainly includes aminosalicylic acid preparations, glucocorticosteroids, and immunosuppressants [6], etc. However, such treatment inevitably causes various side effects and aggravate patients’ injuries. The use of natural products for the treatment of UC is a promising alternative to traditional therapies. Polysaccharides have received widespread attention as an important class of active substances, and polysaccharides have a variety of biological activities such as good antioxidant, anti-inflammatory, and immunomodulatory properties [7]. At the same time, polysaccharides also have the advantages of safety and non-toxicity, good therapeutic efficacy, and biocompatibility.

Billions of different gut microorganisms live in the human gastrointestinal system. A thorough investigation of the intricate relationships between the human gut microbiota and dietary polysaccharides could help to develop the next generation of prebiotics derived from natural sources. At the same time, the gut microbiota is an important part of the intestinal mucosal barrier. In recent years, marine polysaccharides have been increasingly studied for improving gut health by regulating the gut microbiota [8,9]. Algae-derived fucoidan has been shown to be effective in preventing the negative effects of antibiotics on the gut microbiota and colon health [10], while L-fucose accelerates intestinal epithelial development by promoting propionic acid metabolism associated with *Akkermansia* [11]. *Apostichopus japonicus* is a high-quality sea cucumber that contains polysaccharides, active peptides, saponins, phospholipids, and other active ingredients. Aj-FUC is a branched fucoidan composed of a novel pentasaccharide repeating unit [12], which possesses a range of physiological properties including antioxidant, hypolipidemic, and antitumor. Lifestyle and dietary changes may characterize future disease prevention strategies, and we have not yet investigated how the polysaccharides rich in *Apostichopus japonicus*, a high-quality diet, may have a preventive effect on UC. Therefore, this study has the purpose of investigating the efficacy of Aj-FUC in relieving colitis and its potential mechanisms. Meanwhile, through in vitro simulated fermentation experiments, it provides richer evidence for the prebiotic effects of Aj-FUC on human intestinal flora. It not only provides more possible solution strategies for people with impaired intestinal health, but also provides a theoretical basis for the development of fucoidan-related products.

## 2. Materials and Methods

### 2.1. Material and Reagents

*Apostichopus japonicus* was purchased from Qingdao Fisherman’s Pride Sea Cucumber Treasures Co. Dextran sulfate sodium salt (DSS, 36–50 kDa) was obtained from Dalian Meilun Biotechnology Co., Ltd. (Dalian, China). The fecal occult blood kit was provided by Abbott Laboratories (Shanghai, China). ELISA kits for IL-6, IL-1β, and TNF-α were provided by Elisa Bio-technology (Elisa Biotech, Shanghai, China). Omni-Eazy^TM^ one-step PAGE gel preparation kit was provided by Shanghai Epizyme Biomedical Technology (Shanghai, China). RT-qPCR primers were provided by Bioengineering Co., Ltd. (Shanghai, China). Antibodies for ZO-1, and occludin were obtained from Servicebio (Wuhan, China).

### 2.2. Extraction and Structural Characterization of Aj-FUC

#### 2.2.1. Extraction of Aj-FUC

The extraction of Aj-FUC was based on the method of Chang et al. [13]. Specifically, the weighed, dried *Apostichopus japonicus* powder was added to a buffer containing 100 mM acetic acid–sodium acetate, 5 mM disodium ethylenediaminetetraacetic acid, 5 mM L-cysteine at pH 6.0, and enzymatically digested in 0.1-fold papain at 60 °C for 12 h. After the inactivation of the enzyme, the powder was subjected to two alcoholic precipitations with 95% ethanol, then dialyzed and lyophilized to collect Aj-FUC.

#### 2.2.2. Molecular Weight Analysis

The molecular weight of Aj-FUC was determined according to the method described previously [14]. Briefly, the Aj-FUC solution was precisely configured at a concentration of 1 mg/mL, filtered through a 0.22 μm membrane and the results were analyzed by an HPLC1100-18 Angle Laser Scanner with the following conditions: chromatographic column—TSKgel super AW4000 (6.0 mm × 150 mm), column temperature—40 °C, mobile phase—0.2 M NaCl, flow rate—0.5 mL/min, loading volume—100 μL.

#### 2.2.3. Analysis of Monosaccharide Composition

The monosaccharide composition of Aj-FUC was analyzed by PMP-HPLC using an Agilent 12600 system. Briefly, the Aj-FUC was dissolved as a 10 mg/mL solution, and 200 μL of the sample was added into an ampoule, 1 mL of 2 mol/L trifluoroacetic acid solution was added, and the ampoule was filled with nitrogen and sealed. The sample was then digested in an oven at 110 °C for 4 h. After removing the sample, the trifluoroacetic acid was dried in a water bath at 60 °C with air and then adjusted to neutrality by adding sodium hydroxide solution. Next, 70 μL of the sample was taken and mixed with 10 μL of the internal standard for PMP derivatization, followed by the up-sampling operation. The standards used in this study contained seven monosaccharides, mannose (Man), glucosamine (GlcN), D-glucuronic acid (GlcUA), galactosamine hydrochloride (GalN), glucose (Glc), galactose (Gal), and fucose (Fuc).

#### 2.2.4. UV–Vis Absorption Spectroscopy and Fourier-Transform Infrared (FTIR) Spectra Analysis

Aj-FUC was prepared as a 1 mg/mL solution and then scanned using a UV–visible spectrophotometer analyzer (UV-2802, UNICO, Shanghai, China) in the wavelength range of 200–600 nm to obtain the UV spectrogram of Aj-FUC. The appropriate amount of each component of dried Aj-FUC was mixed with KBr and pressed into tablets, which were then scanned and analyzed by a Fourier Transform Infrared Spectrometer (IS-10, Nicolet, Waltham, MA, USA) in the range of 400–4000 cm^−1^ for FTIR spectra.

### 2.3. Animals and Experimental Design

Thirty-six SPF-grade male C57BL/6 mice (18–20 g, 6 weeks old) were acquired from Vital River Laboratories (Beijing, China). They were kept in a specific room (temperature: 24 ± 1 °C, humidity: 55 ± 15 °C, day/night cycle of 12 h) with full availability of food and water. The Ocean University of China’s Animal Ethics Committee gave its approval for the animal research (Approved Protocol No. SPXY2023090710).

Following a 7-day acclimatization period, the mice were allocated into six distinct groups (*n* = 6): Normal control group (N), low-dose Aj-FUC control group (100 mg/kg bw, LF), high-dose Aj-FUC control group (300 mg/kg bw, HF), DSS-induced model group (M), low-dose Aj-FUC treated DSS group (100 mg/kg bw, MLF), and high-dose Aj-FUC treated DSS group (300 mg/kg bw, MHF). The DSS modeling period lasted 15–21 days, while the entire experimental time was 0–24 days. During the modeling period, 2.5% DSS solution was prepared by dissolving 2.5 g DSS in 100 mL of distilled water. Mice in the model group were given 2.5% DSS solution for 7 days from day 15 to day 21, and 200 μL of ultrapure water was administered as a control during the duration of the experiment. While mice in the MLF and MHF group were given 2.5% DSS solution for 7 days from day 15 to day 21, mice in the LF, HF, MLF, and MHF group were gavaged with the corresponding dose of Aj-FUC throughout the experimental period. The mice were then euthanized on day 25. The 1 cm distal colon tissues were fixed in 4% paraformaldehyde solution and examined histopathologically. Serum, colon tissue, and intestinal contents were collected from all mice and stored in a refrigerator at −80 °C for further analysis.

### 2.4. Evaluation of Colitis

The mice were monitored every day after the DSS colitis model was started, and their body weight and fecal condition were noted. Fecal occult blood was measured using a fecal occult blood kit (Shanghai, China). Disease activity index (DAI) scores were calculated based on weight loss, fecal status, and fecal occult blood (Table S1).

### 2.5. Histological Analysis

A 4% paraformaldehyde solution was used to fix around 1 cm of distal colon tissue for 48 h, after which the tissue was dehydrated, embedded, and sectioned, then stained with hematoxylin–eosin (HE) and with alcian blue–periodic acid–Schiff (AB-PAS), and observed and photographed under an electric fluorescence microscope; the HE-stained sections were scored histopathologically. The scoring criteria were referenced from others [15] and are shown in Table S2.

### 2.6. Biochemical Analysis

The mice colon tissues were ground in saline and then centrifuged at 3000× *g* for 10 min to obtain the supernatant. The levels of IL-6, IL-1β, and TNF-α in mice tissues were measured rigorously in accordance with the ELISA kit’s instructions (Shanghai, China).

### 2.7. RT-qPCR Analysis

RNA was extracted from mice colon tissues using Trizol reagent (Thermo Fisher, Shanghai, China), and the RNA was reverse transcribed to form cDNA according to the instructions of the All-in-One 5× RT MasterMix kit. A real-time PCR device (Bio-Rad, Hercules, CA, USA) was used to analyze the genes’ mRNA levels in accordance with the BlasTaq^TM^2 × qPCR MasterMix kit’s instructions (Abmgood, Shanghai, China). Table S3 displays the precise primer sequences used in the experimental tests, and the $2^{-∆∆CT}$ technique was ultimately used to compute the findings.

### 2.8. Western Blotting Analysis

Colon tissue proteins were extracted using RIPA lysate (Solarbio, Beijing, China) containing PMSF (G-CLONE, Bejing, China) and phosphatase inhibitor (Sangon, Shanghai, China). A BCA kit (Epizyme, Shanghai, China) was then used to measure the isolated proteins’ concentration. The following stages provide a summary of the protein blotting procedure [16]. Gel blocks were created to use the Omni-Easy^TM^ One-Step PAGE Gel Rapid Preparation Kit (Epizyme, Shanghai, China) and poured into the electrophoresis buffer after the gels solidified. Then marker and each group of protein samples were added into the sample wells and run at 100 V for 2 h. Protein blotting was transferred using the wet-transfer-to-transfer membrane method. After transfer, the PVDF membrane (Millipore) was closed for 2 h. Then, the PVDF membrane was incubated with primary antibody overnight at 4 °C. After the secondary antibody incubation on the next day, the PVDF membrane was placed in the ECL color development solution (ABclonal, Wuhan, China) and photographed using an imaging system (Tanon, Shanghai, China). It was sufficient to select the appropriate bands according to the degree of exposure.

Primary antibody ZO-1 (dilution 1:2000), primary antibody occludin (dilution 1:2000) were purchased from Sevier (Servicebio, Wuhan, China), and the supplier of GAPDH (dilution 1:2000) was ABclonal (ABclonal, Wuhan, China). The antibody diluent (Epizyme, Shanghai, China) was used to dilute each antibody.

### 2.9. Short Chain Fatty Acids (SCFAs) Assay

Gas chromatography (GC) was used to determine the SCFA content in cecum contents using a previously described method with a few changes [17]. Before being homogenized, each sample of thawed cecum content was mixed with 600 μL of ultrapure water. The homogenate was vortexed after being mixed with 25 μL of 50% sulfuric acid. Following a 10 min centrifugation at 5000× *g*, 300 μL of supernatant was obtained from the samples. This was then mixed with 150 μL of anhydrous ether and 15 μL of the internal standard, 2-ethylbutyric acid. After sufficient vortexing and mixing, the sample was centrifuged at 10,000× *g* and the upper ether layer was aspirated into a liquid phase vial using a 1 mL syringe. The Agilent 6890 system was used for specific analyses.

### 2.10. 16S rRNA Gene Sequencing

First, total fecal DNA from mice was obtained using a fecal DNA extraction kit. The purity of the extracted fecal DNA was determined using Nanodrop 2000 [18]. Next, utilizing the current experimental technique, the V3–V4 region of the 16S rRNA gene was chosen for replication., and the library was constructed and up-sequenced.

### 2.11. In Vitro Fermentation of Aj-FUC by Human Gut Microbiota

The in vitro fecal fermentation medium was configured as follows [19]. Briefly, 10.0 g of casein peptone, 2.5 g of yeast extract, 0.001 g of bladed azure, 0.45 g of dipotassium hydrogen phosphate, 0.45 g of potassium dihydrogen phosphate, 0.9 g of sodium chloride, 4.0 g of sodium bicarbonate, 0.044 g of magnesium sulfate, 0.09 g of calcium chloride, 1.0 g of L-cysteine, 0.05 g of hemoglobin chloride, and 2.0 mL of Tween 80 were dissolved in 1.0 L distilled water, the pH adjusted with HCl, and autoclaved after nitrogen blowing to obtain the basal medium. Fecal samples from four healthy donors were collected for the normal control group. Additionally, fresh fecal specimens from IBD patients were supplied by the Department of Gastroenterology at The First Affiliated Hospital of Qingdao University (Qingdao, China). After collection, 10% (*w*/*v*) fecal homogenate was obtained by mixing equal weights of each feces from healthy volunteers and IBD patients with autoclaved PBS. Then 1.0 mL of feces was inoculated into 9.0 mL of basal medium or basal medium containing 10 g/L glucose or 10 g/L Aj-FUC. The healthy fecal samples were named Ctrl, Glu, and Fuc group, and the IBD fecal samples named Ctrl-IBD, Glu-IBD, and Fuc-IBD group. The fermentation solution was anaerobically fermented at 37 °C for 48 h, after which the fermentation broth was collected for 16SrRNA sequencing.

### 2.12. Statistical Analysis

In this article, all data were displayed as mean ± standard deviation (mean ± SEM). One-way ANOVA and post hoc LSD were performed by GraphPad Prism (version 9.5, GraphPad Software Inc., La Jolla, CA, USA). *p*-values < 0.05 indicate statistically significant variance.

## 3. Results

### 3.1. Structural Characterization of Aj-FUC

**Figure 1:**
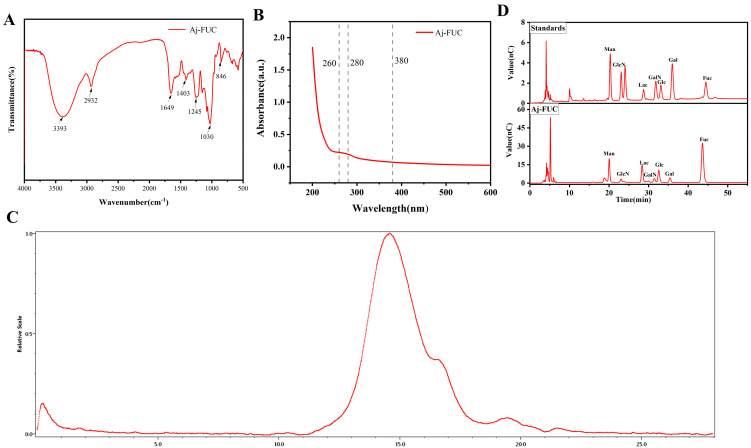
Structural characterization of Aj-FUC. (**A**) FT-IR spectra; (**B**) UV spectrum; (**C**) molecular weight measurement; (**D**) monosaccharide composition.

The FTIR spectrum of Aj-FUC is shown in Figure 1A, with the telescopic vibration peaks of hydroxyl O-H and saccharide methyl-CH2 at 3393 cm^−1^ and 2932 cm^−1^, the telescopic vibration peak of sulfate group S=O at 1245 cm^−1^, and the absorption peak at 846 cm^−1^, which is the characteristic absorption peak of the α-glycosidic bond, and the special absorption of the C-O-S vibration. This indicates that the extract of the sample was a polysaccharide containing sulfate with an α-glycosidic bond as the main component. The UV absorption spectra of Aj-FUC are shown in Figure 1B; there were no visible absorption peaks at 260 nm, 280 nm, and 380 nm, indicating that the extracted polysaccharide was free of proteins and pigments, and the purity of the polysaccharide was high. The molecular weight of Aj-FUC was 774.2 ± 1.34 kDa, as can be seen in Figure 1C. The data of the monosaccharide composition are shown in Figure 1D, which indicated that the Aj-FUC mainly consisted of Fuc, Glc, Man, GalN, Gal, and GlcN, with a molar ratio of 0.51:0.2:0.15:0.06:0.04:0.04.

### 3.2. Aj-FUC Treatment Attenuates Pathological Histologic Damage in Colitis Mice

**Figure 2:**
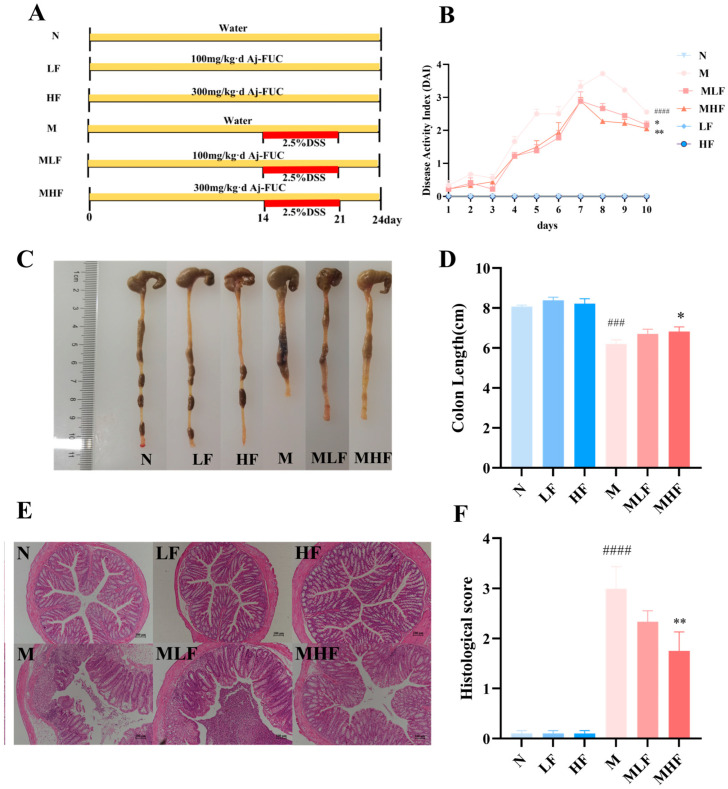
Effect of Aj-FUC on basic indicators of pathologic histology in mice with DSS-induced colitis. (**A**) Experimental design; (**B**) disease activity index (DAI); (**C**) representative colonic images of mice; (**D**) length of mice colon; (**E**) HE stained section of mice colon; (**F**) pathohistological scores. ### *p* < 0.001, #### *p* < 0.0001 vs. N; * *p* < 0.05, ** *p* < 0.01 vs. M.

To evaluate the alleviating efficacy of Aj-FUC on DSS-induced colitis, after one week of acclimatization culture, mice were gavaged with Aj-FUC at a dose of 100 mg/kg bw or 300 mg/kg bw according to the subgroup for 14 days. Subsequently, 2.5% DSS was added to the drinking water of mice for one week to induce acute colitis as shown in Figure 2A. DSS is a sulfated polysaccharide synthesized from sucrose, and an ulcerative colitis model can be simulated by mice drinking a certain concentration of DSS ad libitum. DSS induces toxic disruption of the colonic epithelial barrier, damaging the integrity of the colon, which in turn induces a range of inflammatory responses, and is a commonly used anti-inflammatory drug model for the study of colitis in humans. During the modeling period, mice were examined daily for changes in body weight as well as DAI scores. The mice were executed on day 25 to assess the colonic lesions.

The DAI score reflects the severity of colonic injury, and the length of the colon is inversely proportional to the severity of colitis. Throughout the modelling period in this study, the mice in group N remained healthy, and mice in group M developed diarrhea and blood in the stool due to the consumption of 2.5% DSS water, with significantly higher DAI scores and significantly shorter colon lengths (Figure 2B–D), suggesting that colitis had been successfully induced. Compared with group M, Aj-FUC intervention reversed the shortening of colon length caused by DSS, and mice in the MHF group had considerably longer colons than those in group M (*p* < 0.05). Meanwhile, the DAI score was significantly reduced, and the phenomena of diarrhea and blood in stool were improved.

In group N mice, the colonic epithelial cells were neatly and tightly arranged, the number of goblet cells was normal, and the crypt structure was clear. The mice in group M developed ulcers in the colonic mucosa, with missing goblet cells and disrupted crypt structure, accompanied by severe inflammatory cell infiltration. In the MLF and MHF-treated group with prophylactic ingestion of Aj-FUC, the inflammatory cell infiltration was alleviated, with some restoration of goblet cells and crypt structures, and the pathohistological scores were considerably lower than those of the M group (Figure 2E,F). These findings suggest that prophylactic supplementation with Aj-FUC can attenuate the pathohistological damage in colitis mice.

### 3.3. Aj-FUC Treatment Modulates the Inflammatory Response in Mice with Colitis

**Figure 3:**
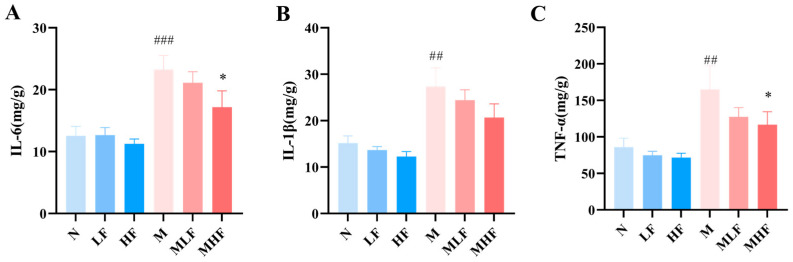
Effect of Aj-FUC on tissue inflammatory factors in mice with DSS-induced colitis. (**A**) IL-6; (**B**) IL-1β; (**C**) TNF-α. ## *p* < 0.01, ### *p* < 0.001 vs. N; * *p* < 0.05 vs. M.

To assess the effect of Aj-FUC on colitis inflammation, the ELISA kit was used to evaluate the expression levels of different inflammatory factors in the colon tissues of mice (Elisa Biotech, Shanghai, China). The findings demonstrated that group M had significantly higher levels of inflammatory markers, like IL-6, IL-1β, and TNF-α (Figure 3A–C), than group N. Compared with group M, Aj-FUC pretreatment reduced the levels of IL-6, IL-1β, and TNF-α, while the dose of 300 mg/kg bw Aj-FUC treatment was significant for the reduction of IL-6 (*p* < 0.05) and TNF-α (*p* < 0.05). These findings indicate that taking Aj-FUC supplements may help reduce the inflammation linked to UC.

### 3.4. Aj-FUC Treatment Modulates the Intestinal Barrier in Mice with Colitis

**Figure 4:**
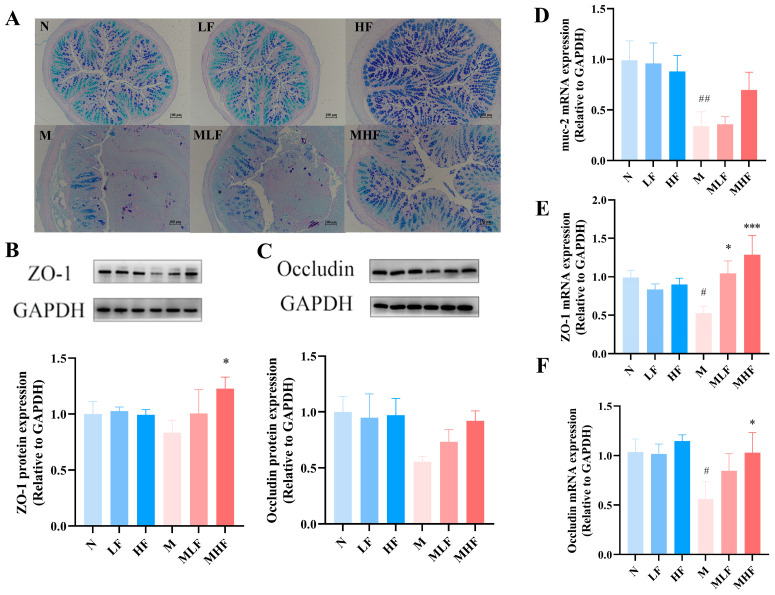
Effect of Aj-FUC on DSS-induced intestinal mucosal barrier in mice. (**A**) Pictures of AB-PAS-stained colon sections; (**B**) the protein levels of ZO-1; (**C**) the protein levels of occludin; (**D**) the mRNA levels of muc-2; (**E**) the mRNA levels of ZO-1; (**F**) the mRNA levels of occludin. # *p* < 0.05, ## *p* < 0.01 vs. N; * *p* < 0.05, *** *p* < 0.001 vs. M.

The initial line of defense between the intestinal system and the outside world is the intestinal mucosal barrier, which is crucial for preventing pathogen invasion and toxin absorption [20]. To assess the impact of Aj-FUC on the colonic mucosal barrier, the quantity of goblet cells in the intestinal tissues was observed by AB-PAS staining. The results are shown in Figure 4A. The N, LF, and HF group exhibited abundant mucin on the surface of the colonic epithelium and within the goblet cells. In contrast, the mucus layer of group M was severely damaged, with significantly fewer goblet cells and significantly fewer mucin-rich areas compared with group N. Compared with group M, the goblet cells were noticeably higher in the MHF group of mice prophylactically supplemented with Aj-FUC. The protein expression levels of ZO-1, and occludin were then measured to assess the intestinal barrier function. As shown in Figure 4B,C, the protein expression levels of ZO-1 and occludin were reduced in group M compared with group N. Preventive supplementation with Aj-FUC upregulated the protein expression levels of ZO-1 and occludin, and the upregulation of the protein expression of ZO-1 was significant (*p* < 0.05). The mRNA expression levels of muc-2, ZO-1, and occludin were further determined, and MHF could increase the expression levels of the genes compared with group M. The increase was significant for ZO-1 (*p* < 0.001), and occludin (*p* < 0.05) (Figure 4D–F). These results suggest that prophylactic supplementation of Aj-FUC can modulate the intestinal mucosal barrier in mice.

### 3.5. Aj-FUC Treatment Promotes the Production of SCFAs in Colitis Mice

**Figure 5:**
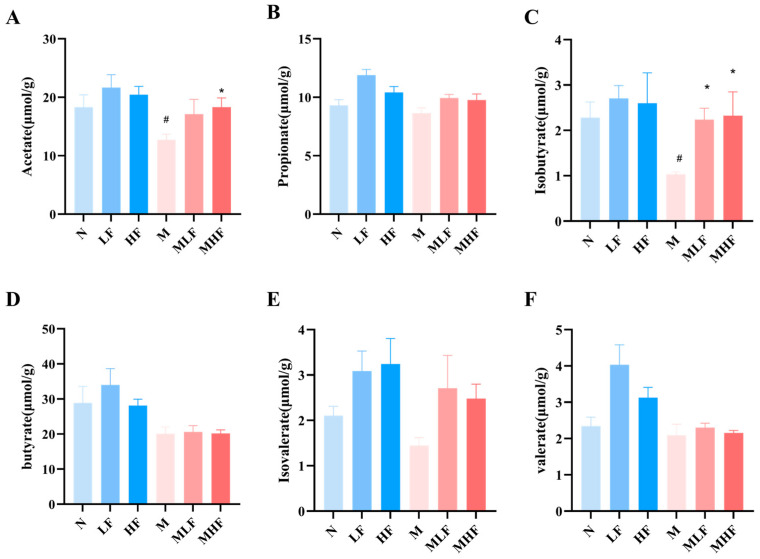
Effect of Aj-FUC treatment on SCFA concentration in DSS-induced mice in the contents of the cecum: (**A**) acetate; (**B**) propionate; (**C**) isobutyrate; (**D**) butyrate; (**E**) isovalerate; (**F**) valerate. # *p* < 0.05 vs. N; * *p* < 0.05 vs. M.

The intestinal microbiota of the gastrointestinal tract ferments indigestible carbohydrates to create SCFAs, which are crucial for preserving the body’s homeostasis and intestinal barrier integrity [21]. Therefore, the content of SCFAs in the contents of the cecum was further analyzed and determined. As shown in Figure 5A–F, the content of acetate, propionate, butyrate, isobutyrate, valerate, and isovalerate were reduced in group M compared with group N, especially acetate (*p* < 0.05) and isobutyrate (*p* < 0.05). The production of SCFAs showed an increased trend in the MLF and MHF group supplemented with Aj-FUC compared with the M group, and the MHF group had significantly increased levels of acetate (*p* < 0.05) and isobutyrate (*p* < 0.05). These findings support that prophylactic supplementation with Aj-FUC may modulate the metabolism of the gut microbiota to attenuate DSS-induced colitis.

### 3.6. Aj-FUC Treatment Regulates Intestinal Microbiota in Mice with Colitis

**Figure 6:**
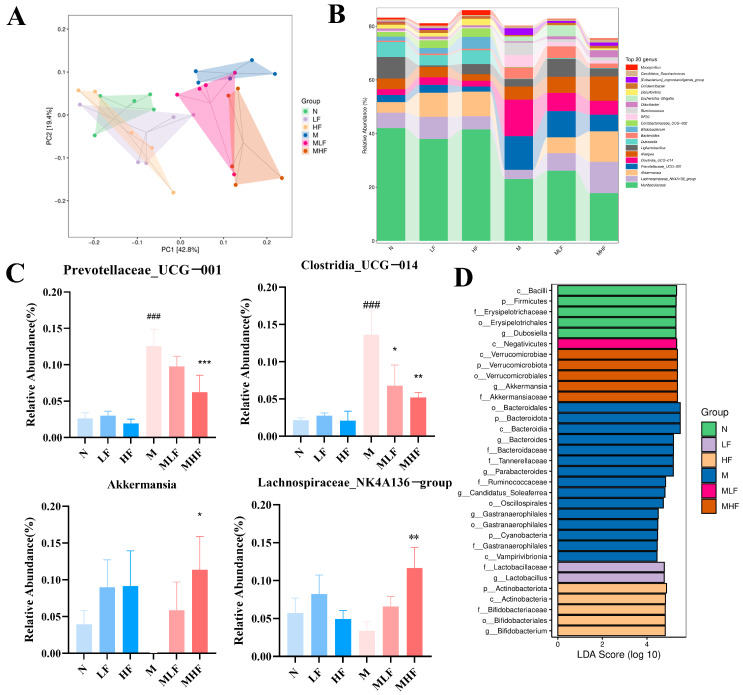
Regulation of intestinal microbiota in mice with DSS-induced colitis by Aj-FUC. (**A**) Principal Coordinate Analysis; (**B**) effect of Aj-FUC on the composition and structure of intestinal microbiota in mice; (**C**) relative abundance of Akkermansia, Lachnospiraceae_NK4A136_group, Prevotellaceae UCG-00l, Clostridia UCG-014; (**D**) LDA scores based on LEfSe analysis. ### *p* < 0.001 vs. N; * *p* < 0.05, ** *p* < 0.01, *** *p* < 0.001 vs. M.

The effects of Aj-FUC administration on the composition and structure of the gut microbiota in mice with colitis were examined because of the vital role that gut microbiota plays in preserving intestinal homeostasis. Principal coordinate analysis (PCA) is shown in Figure 6A; the gut microbiota diversity of mice in group M was significantly different from that of group N, while Aj-FUC intervention prevented the bias of the intestinal microbiota towards group N. Figure 6B,C shows the structural composition of the intestinal microbiota of the mice in each group at the genus level, and compared with group N, the DSS treatment significantly increased the Clostridia_UCG-014 (*p* < 0.001), Prevotellaceae_UCG-001 (*p* < 0.001) and other pathogenic bacteria. On the other hand, supplementation with Aj-FUC markedly raised the percentage of beneficial bacteria such as Akkermansia (*p* < 0.05), Lachnospiraceae_NK4A136_group (*p* < 0.01) compared with group M.

Linear discriminant analysis effect size (LEfSe) analysis enables the comparison among multiple groups and pinpoints species that exhibit notable differences in abundance across these groups. As shown in Figure 6D, mice supplemented with Aj-FUC exhibited enrichment of Akkermansia, Lactobacillus, and Bifidobacterium. The findings suggest that the gut microbiota benefited from the administration of Aj-FUC supplements, which promoted a rise in good bacteria and a fall in bad bacteria.

### 3.7. Aj-FUC Fermentation Contributes to the Balance of Human Intestinal Microorganisms and Improves Intestinal Microbiota in Colitis Patients

**Figure 7:**
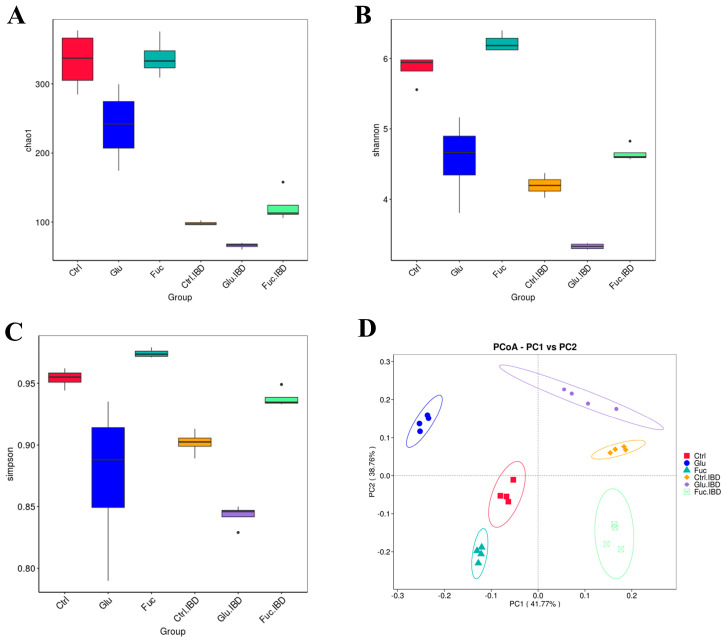
Aj-FUC alters human gut microbiota diversity through in vitro fermentation. (**A**) Chao1 index; (**B**) Shannon index; (**C**) Simpson index; (**D**) principal coordinate analysis.

It has been shown that the natural gut microbiota of mice and humans are very different, with only 4% of human gut microbes having 95% identity with mice gut microbiota [22]. Therefore, the effects of Aj-FUC on regulating the gut microbiota in healthy and IBD patients were further explored by in vitro fecal simulated fermentation. In order to assess the ecological characteristics of the bacterial community of different substrates after 48 h of in vitro fermentation, the α-diversity index of bacteria was measured, and the results are shown in Figure 7A–C. Species richness and diversity of the gut microbiota were reduced after glucose fermentation compared with Ctrl and Ctrl-IBD without added carbon source, but Aj-FUC fermentation could largely maintain high species richness and diversity. The impact of various substrates on the structure and composition of the gut microbiota were assessed by β-diversity analysis, and the results are shown in Figure 7D. The structure and composition of the gut microbiota were significantly different in different groups, and the IBD gut microbiota (Ctrl-IBD, Glu-IBD, Fuc-IBD) had a different structure compared with the healthy gut microbiota (Ctrl, Glu, Fuc). In particular, the Fuc-IBD group moved away from the IBD group (Ctrl-IBD and Glu-IBD) and tended towards the Ctrl group, indicating that the Aj-FUC intervention may encourage structural alterations in the IBD gut microbiota that resemble those of the healthy group.

**Figure 8:**
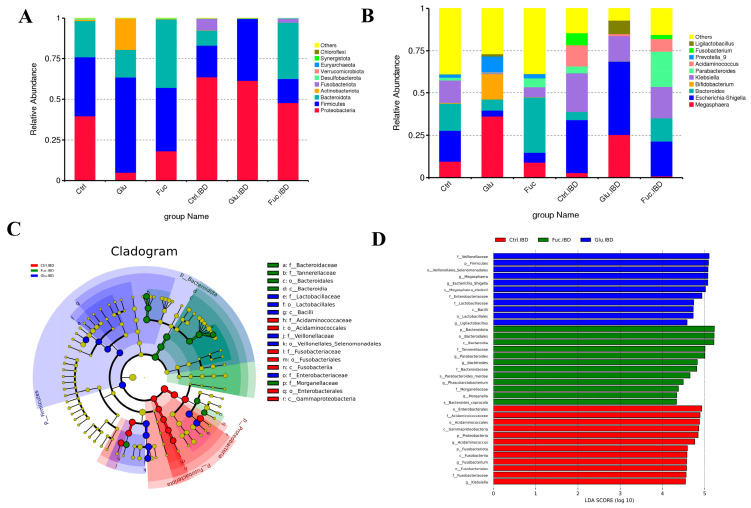
Alterations in human gut microbiota after 48 h of Aj-FUC fermentation. (**A**) Relative abundance of microbial communities at the phylum level; (**B**) relative abundance of microbial communities at the genus level; (**C**) description of LEfSe analysis for microbial characterization; (**D**) LDA scores based on LEfSe analysis.

Microbial characterization at the phylum level is shown in Figure 8A; the gut microbiota composition of the healthy and IBD group consisted mainly of Proteobacteria, Firmicutes, and Bacteroidota, with an increased abundance of Proteobacteria and a decreased abundance of Bacteroidota in the IBD group. After glucose fermentation, the abundance of Firmicutes continued to increase and the abundance of Bacteroidota continued to decrease. In contrast, Aj-FUC fermentation enhanced the abundance of Bacteroidota while decreasing that of Firmicutes, thus decreasing the Firmicutes/Bacteroidota ratio and causing the IBD group to converge to a healthy group. Microbial characterization at the genus level is shown in Figure 8B, with more Escherichia–Shigella and Klebsiella in the IBD group. After glucose fermentation, the feces of the IBD population showed a further increase in Escherichia–Shigella, with the relative abundance of Bacteroides and Parabacteroides further decreased. On the contrary, after fermentation with Aj-FUC, Escherichia–Shigella decreased in the feces of the IBD group, while Bacteroides, Parabaacteroides, etc., significantly increased. The results of LEfSe analysis showed (Figure 8C,D) that pathogenic bacteria such as Escherichia–Shigella and Enterobacteriacea were enriched in Glu-IBD, whereas anti-inflammatory bacteria such as Bacteroides and Parabacteroides were enriched in the Fuc-IBD group after fermentation of Aj-FUC. These findings suggest that Aj-FUC fermentation has the potential to influence the fecal microbiota of both healthy individuals and IBD patients, and that Aj-FUC acts as a modulator to improve the gut environment of IBD patients.

## 4. Discussion

In the past decade, dietary interventions have become the mainstay of IBD prevention and treatment [23]. *Apostichopus japonicus* is an important marine farmed species in China with high nutritional and medicinal values [24]. In this study, the effects of Aj-FUC on DSS-induced colitis in mice was explored, focusing on disease severity, the intestinal barrier, and gut microbiota.

Fucoidan sulfate is a polysaccharide rich in sulfate groups obtained from marine brown algae and marine invertebrates. In contrast to the structure of fucoidan sulfate from seaweed sources, Aj-FUC is a linear polysaccharide consisting of L-fucose linked by α-1,3-glycosidic bonds [25]. In this study, mice were given Aj-FUC orally for 14 days and then DSS colitis was induced for 7 days. Supplementation with Aj-FUC attenuated the symptoms of colitis in mice, including reduced DAI scores, histological damage, enhanced intestinal barrier, and improved gut microbiota.

The initial line of defense between the intestinal system and the outside world is the intestinal mucosal barrier [26]. In recent years, one of the main contributing factors to the pathophysiology of IBD was identified as deterioration of the intestinal mucosal barrier function [27]. As the primary means of communication between intestinal mucosal epithelial cells, tight junctions are crucial for preserving the normal operation and structural integrity of the intestinal mucosal barrier apparatus. ZO-1 [28] and occludin [29] are the main components of tight junctions. Reduced activity or down-regulation of their expression impacts the development of intercellular tight junctions and hinders the intestinal mucosa’s crucial defense barrier function. Polysaccharides, as a nutrient widely found in plants and animals, have been shown to have the ability to regulate the intestinal mucosal barrier. In this study, Aj-FUC was found to increase the expression of ZO-1, occludin, and alleviate the intestinal barrier damage caused by UC.

A vast and varied collection of bacteria are parasitized in the human gut, and the gut microbiota plays a vital role in maintaining human health [30]. There is growing evidence that UC is intimately associated with gut microbial dysbiosis [31]. The most obvious change in patients with UC is a rise in pathogenic bacteria and a fall in helpful bacteria, as compared with healthy organisms [32]. Previous studies demonstrated that colonization of pathogenic bacteria, such as *Clostridia UCG014_norank*, *Prevotellaceae UCG-001* members, exacerbates intestinal inflammation in mice [33,34]. In contrast, *Faecalibacterium prausnitzii* and *Akkermansia* [35] have anti-inflammatory effects, enhance intestinal epithelial cell integrity, and maintain intestinal homeostasis. However, the abundance of these bacteria is reduced in UC patients. This was confirmed by the results, in that the species composition of the gut microbiota of mice in group M formed a significant difference from group N, with *Akkermansia*, *Lachnospiraceae_NK4A136_group* being significantly lower. The abundance of these bacterium was significantly increased in mice ingesting Aj-FUC. These bacteria are also core genera for the production of the beneficial intestinal metabolite SCFAs [21].

*Akkermansia* is a mucus layer-degrading member of the human and mouse gut microbiota [36] and is a promising candidate for a next-generation probiotic [37]. *Akkermansia* has been reported to be effective in modulating human health, including metabolic regulation, immunomodulation, and intestinal health protection [38]. Several studies showed that *Akkermansia* can alleviate colitis by modulating the intestinal barrier [35]. The findings of the current study are in line with this, in that supplementation with Aj-FUC can elevate the abundance of *Akkermansia*. Colonization of probiotics increases the opportunity for interaction with the host and increases their transit time in the gut to exert their beneficial effects [39]. The ability of *Akkermansia* to colonize is closely related to its effectiveness in alleviating intestinal problems. To determine the effect of Aj-FUC on the colonization of *Akkermansia* in the gut, *Akkermansia* labelled with fluorescence was administered to mice alone or co-administered with Aj-FUC; the results are shown in Figure S1. We found that fluorescence was stronger in the intestines of mice co-administered with Aj-FUC, meaning that Aj-FUC promotes stable colonization of *Akkermansia* and provides nutrient ecological niches.

Meanwhile, previous studies focused on mouse models, but the gut microbiota of mice and humans are somewhat different, and the fermentation process of Aj-FUC by the microbiota in healthy individuals or those with IBD has yet to be explored. It was found that *Dictyophora indusiata* polysaccharides could increase the abundance of beneficial bacteria through in vitro digestion and act as prebiotics [40]. The polysaccharides extracted from Fuzhuan brick tea were discovered to alter the composition and structure of the gut microbiota in IBD patients through in vitro fecal fermentation studies [41]. In this study, the modulatory effect of Aj-FUC on the human gut microbiota, especially the gut microbiota of IBD patients, was demonstrated for the first time by in vitro simulation of fecal fermentation and high-throughput sequencing of 16Sr RNA. Differences in gut microbiota composition between healthy individuals and IBD patients have been widely reported. The gut microbiota of IBD patients showed lower levels of *Bacteroidota* and relatively higher abundance of *Firmicutes* and *Proteobacteria* [42]. In this study, pathogenic bacteria such as *Escherichia–Shigella*, *Enterobacteriacea*, and *Fusobacterium* were enriched in IBD patients. *Proteobacteria* containing a variety of harmful pathogens, particularly *Escherichia–Shigella* and *Enterobacteriacea* were found at higher levels in IBD patients. This is considered to be the main microbial signature of gut dysbiosis in IBD patients [41]. Fermentation of Aj-FUC resulted in significantly elevated *Bacteroidota* in IBD patients, especially *Bacteroides* and *Parabacteroides*, which have been shown to have potent anti-inflammatory effects [43]. Therefore, Aj-FUC can be used as a modulator to improve the gut microbiota in IBD patients, which lays the scientific foundation for the next treatment of IBD disease.

Intestinal homeostasis consists of the intestinal mucosal barrier, the intestinal environment, and its metabolites, which interact with each other to form a dynamic balance. The intestinal mucosal barrier plays a crucial role in maintaining gut health, with the intestinal microbiota being a key component. An imbalance in the intestinal flora can result in the disruption of the mucosal barrier [44]. In this study, AJ-FUC not only improved intestinal mucosal injury by increasing the expression of ZO-1 and occludin, but also improved the composition and structure of the intestinal flora, especially increasing the abundance of *Akkermansia*, which has been shown to be an important microorganism in maintaining the intestinal mucosal barrier. Ion channels play an important role in regulating intestinal barrier function [45] as well as inflammatory responses [46], so the improvement of intestinal barrier function by Aj-FUC may also indirectly affect ion channel activity.

In conclusion, these results demonstrate that Aj-FUC can prevent UC by resisting gut mucosal damage, regulating inflammatory factors and the homeostasis of gut microorganisms, and in particular, it has a better proliferative and colonizing effect on the next-generation probiotic *Akkermansia*. At the same time, the effect is improved with increasing concentration within a certain range. Meanwhile, the PICRUST function prediction results showed that in Figure S2, Aj-FUC was found to be related to the metabolic functions of the gut microbiota, especially the carbohydrate metabolism, and amino acid metabolism. The production of either the helpful metabolites that can prevent disease or the detrimental metabolites linked to disease development is one way that the gut microbiota influences human health and disease [47]. After that, it is feasible to gain a deeper comprehension of the constituents within the gut microbiota and its polysaccharide metabolism mechanisms, replenishing the gut with host-beneficial intestinal commensal bacteria and regulating the levels of specific metabolites in the gut. However, the sample size of in vitro simulated fecal fermentation was not very large, and the composition of the gut microbiota varies from one colitis patient to another. Consequently, there is a need for more extensive and meticulous research with increased sample sizes to delve deeper into the specific impacts of Aj-FUC on the human gut microbiota.

## 5. Conclusions

In this research, Aj-FUC was derived from the *Apostichopus japonicus*, and the effect of Aj-FUC on the improvement of UC symptoms was investigated using DSS-induced colitis in mice. The results showed that Aj-FUC effectively improved the symptoms of shortened colon and blood in stool in UC mice, and also regulated the levels of cellular inflammatory factors and improved intestinal mucosal damage. Meanwhile, oral administration of Aj-FUC helped to reorganize the intestinal microbiota and restore the relative abundance of specific bacteria, which promoted the increase of the relative abundance of beneficial bacteria and the enhancement of their colonization ability. Aj-FUC was further found to regulate the gut microbiota structure of IBD patients closer to a healthy population by in vitro fecal simulated fermentation, which can increase the relative abundance of anti-inflammatory related probiotics in the flora. These findings indicate that Aj-FUC has the potential to prevent and assist in the treatment of UC.

## Data Availability

The original contributions presented in this study are included in the Article/Supplementary Material. Further inquiries can be directed to the corresponding author.

## Supplementary Materials

The following supporting information can be downloaded at: https://www.mdpi.com/article/10.3390/foods14040574/s1, Table S1: The criteria for disease activity index (DAI) scores; Table S2: The criteria for histopathology score; Table S3: The primer sequences; Figure S1: Fluorescence images of *Akkermansia* distribution in the mouse intestine after 12 h of gavage of *Akkermansia* (DIR) and *Akkermansia* (DIR)+Aj-FUC; Figure S2: The PICRUST function prediction.
