# Supplementary material for: Investigating the Alleviating Effect of Fucoidan from Apostichopus japonicus on Ulcerative Colitis by Mice Experiments and In Vitro Simulation of Human Fecal Fermentation
Source: Foods. 2025 Feb 9;14(4):574. doi: 10.3390/foods14040574 (PMC11853958; doi:10.3390/foods14040574)

Supplementary data

Table S1. The criteria for Disease Activity Index (DAI) scores.

Score value	Fecal status	Fecal occult blood	Weight loss
0	Normal	None	<1%
1			=1-5%
2	Soft	Blood	=5-10%
3			=10-20 %
4	Diarrhea	Abundant bleeding	>20 %

Table S2. The criteria for histopathology score.

Score value	Extent of inflammation	Depth of injury	Crypt damage	Diseased region
0	None	None	None	None
1	Slight	Mucosa	Small proportion of crypt/epithelium damaged	1-25%
2	Mild	Submucosa	Moderate proportion of crypt/epithelium damaged	26-50%
3	Moderate	Muscular layer	Majority of crypt/epithelium damaged	51-75%
4	Severe	Serosal layer	All crypt/epithelium damaged	76-100%

Table S3. The primer sequences.

Primer name	Primer sequence 5’-3’
Muc-2	Forward: 5'-AGGGCTCGGAACTCCAGAAA- 3'<br>Reverse: 5'-CCAGGGAATCGGTAGACATCG- 3'
ZO-1	Forward: 5'-GCGAACAGAAGGAGCGAGAAGAG- 3'<br>Reverse: 5'-GTGGGCTTTGCGGGCTGAC- 3'
Occludin	Forward: 5'-CAGGTGAATGGGTCACCGAG- 3'<br>Reverse: 5'-CAGGCTCCCAAGATAAGCGA- 3'
GAPDH	Forward:5'-GGTTGTCTCCTGCGACTTCA- 3'<br>Reverse:5'-TGGTCCAGGGTTTCTTACTCC- 3'

Figure S1. Fluorescence images of *Akkermansia* distribution in the mouse intestine after 12h of gavage of *Akkermansia* (DIR) and *Akkermansia* (DIR)+Aj-FUC

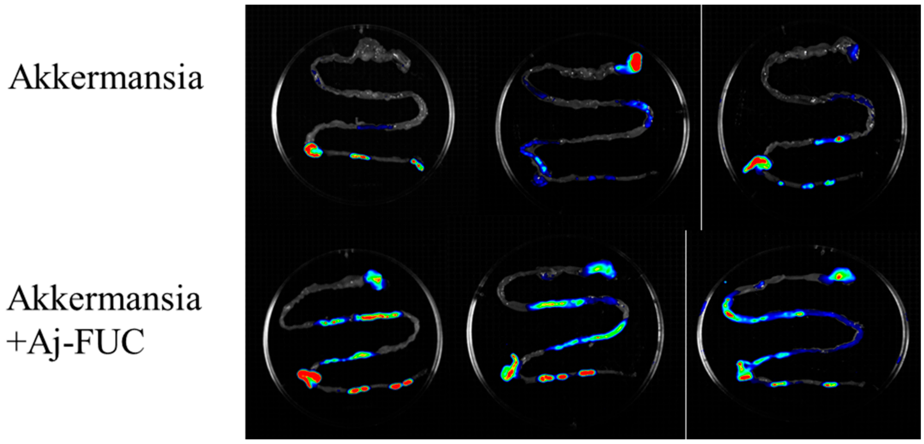

Figure S2. the PICRUST function prediction

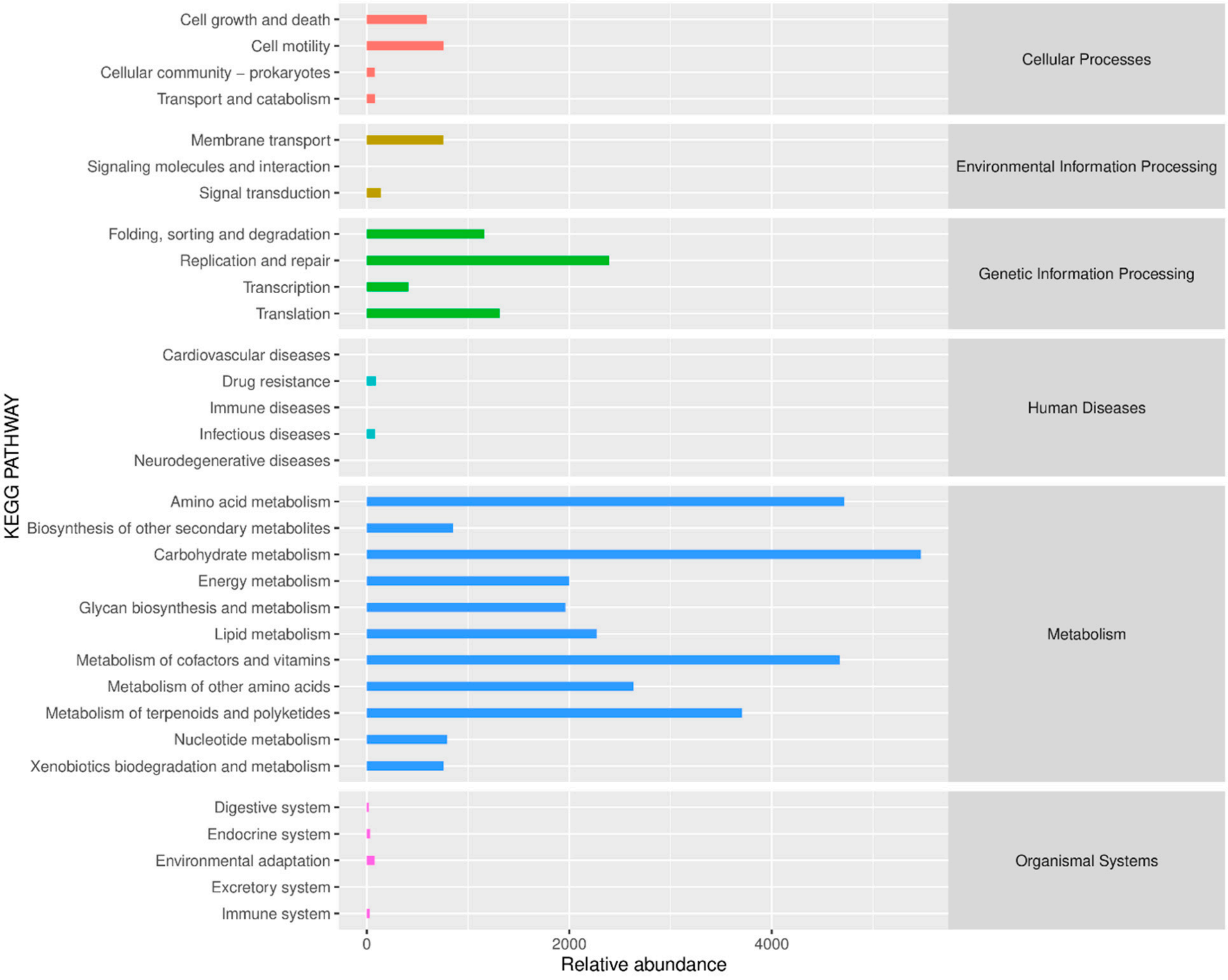

Supplement: Supplementary file 1 [file foods-14-00574-s001.zip › foods-3398283-supplementary.pdf]
